# 569. Cannabis Exposure and Clinical Outcomes in Burn Patients: A Systematic Review

**DOI:** 10.1093/jbcr/irag033.309

**Published:** 2026-04-06

**Authors:** Sara Sheikh-Oleslami, Amir Khorrami, Anthony Papp

**Affiliations:** The University of British Columbia, Vancouver, BC; Vancouver General Hospital BC Burn Unit, Vancouver, BC; Vancouver General Hospital BC Burn Unit, Vancouver, BC

## Abstract

**Introduction:**

Burn injuries remain a leading cause of morbidity and mortality worldwide, with survivors often requiring prolonged hospitalization, complex wound care, and intensive rehabilitation. Concurrently, cannabis use has risen substantially in North America and Europe, with recent data showing cannabis positivity in approximately 16–18% of burn admissions by 2025. Cannabinoids influence pain, immunity, and cardiopulmonary physiology, making their role in burn recovery clinically relevant but poorly defined.

**Methods:**

A systematic review was conducted evaluating cannabis exposure and outcomes in burn patients. MEDLINE, Embase, CINAHL, and Cochrane databases were searched from inception to August 2025. Eligible studies included burn cohorts that reported outcomes stratified by cannabis exposure. Outcomes of interest included mortality, infection, graft success, thromboembolism, ventilation, ICU and hospital length of stay, opioid use, discharge disposition, and readmission.

**Results:**

Of 1009 studies, eight met inclusion criteria, with sample sizes ranging from 64 to over 300 000 patients. Cannabis exposure was identified via toxicology, history, or coding. Across studies, cannabis was not consistently associated with inpatient mortality. Some single-center analyses reported increased opioid and anxiolytic requirements, higher wound infection rates, longer ICU stays, and more reoperations. National database studies found lower adjusted mortality among cannabis-positive patients but higher rates of ventilator-associated pneumonia, venous thromboembolism, and prolonged ICU use. Heterogeneity was pronounced, with polysubstance use, burn severity, and demographic differences influencing outcomes.

**Conclusions:**

Overall, cannabis exposure does not appear to worsen survival in burn patients but may predispose to infectious, respiratory, and thromboembolic complications. The evidence base is limited by retrospective design, inconsistent exposure definitions, and confounding from polysubstance use.

**Applicability of Research to Practice:**

Cannabis positivity at admission should prompt heightened monitoring for complications and tailored pain management. Future prospective, multicenter studies with detailed exposure characterization and long-term follow-up are needed to clarify cannabis’ role in burn recovery and to inform evidence-based analgesic strategies.

**Funding for the study:**

N/A.

